# Reply to the editorial comment from Van der Wall concerning ‘The right ventricle: always normal in normal subjects?’

**DOI:** 10.1007/s12471-015-0683-8

**Published:** 2015-04-08

**Authors:** S. Quick, R.H. Strasser, U. Speiser

**Affiliations:** Klinik für Innere Medizin und Kardiologie, Herzzentrum Dresden Universitätsklinik, Technische Universität Dresden, Fetscherstraße 76, Dresden, 01307 Germany

To the Editor,

We read the comment referring to the challenges in imaging the right ventricle with great interest [1]. The difficulties in distinguishing wall motion abnormalities in healthy subjects from those in diseased patients are very well summarised. The author cites the article by Doesch et al. [2] presenting 20 healthy subjects with CMR-derived measurement of the tricuspid annular plane systolic excursion (TAPSE) with a reference point outside the ventricle (TAPSEout), which might be used for screening right ventricular motion.

We could demonstrate TAPSE in cardiac magnetic resonance (CMR), being a fast and easily obtainable parameter correlating well to volumetric quantification of right ventricular ejection fraction (RVEF), with low interobserver and intraobserver variables. Therefore, we investigated 76 patients (age: 58 ± 17 years) with mean RVEF of 42 ± 14 %, assessed by the standardised slice-summation method. CMR-TAPSE was determined to be 19 ± 6 mm and correlated well in linear regression analysis with volumetric RVEF (*r* = 0.72, *p* < 0.001). However, we could also show that CMR-TAPSE is not only a fast tool in healthy subjects, but it also discriminates well between patients with impaired and normal RVEF. Multiplying CMR-TAPSE measurements by 2.5 leads to values close to the RVEF by volumetry. Furthermore, CMR-TAPSE correlates well with TAPSE determined using transthoracic echocardiography [3].

## Funding

None.
